# The Integration of Artificial Intelligence with Micro–Nano-Systems: Perspectives, Challenges and Future Prospects

**DOI:** 10.3390/mi16030301

**Published:** 2025-03-04

**Authors:** Juvenal Rodríguez-Reséndiz, Marcos Aviles, José M. Álvarez-Alvarado

**Affiliations:** Facultad de Ingeniería, Universidad Autónoma de Querétaro, Querétaro 76010, Mexico; juvenal@uaq.edu.mx

Technological advances have allowed various systems to be developed on a small scale. The computational processing and performance of such systems are, in most cases, more efficient than those of a personal computer [1,2]. This Editorial presents a technical analysis for a Special Issue focused on the challenges and perspectives regarding artificial intelligence in micro–nano-systems. To observe the trend in the topics, a bibliometric network is presented in Figure 1, which shows the main topics of interest that were reported in the published works. The main trends in the areas of biomedicine, energy, the automotive industry and imaging are identified.

In particular, the integration of micro- and nano-systems in energy management and utilization has significantly transformed the efficiency and functionality of devices across a wide range of applications. A prime example is the use of triboelectric nanogenerators, which have emerged as an innovative technology for harvesting environmental energy by converting subtle mechanical movements into usable electricity. This capability is especially valuable for the autonomous operation of portable devices and independent sensors [3]. Likewise, nanomaterials, such as those based on graphene, have shown great energy storage potential by enhancing the capacity and stability of lithium-ion batteries, which is crucial for applications such as consumer electronics and electric vehicles [4]. Similarly, the implementation of nanomaterials in solar cells has significantly increased solar energy absorption and conversion efficiency, thus contributing to the development of more effective renewable energy sources [5]. These advances highlight the fundamental role of micro- and nano-systems in optimizing energy utilization, providing innovative solutions to address contemporary energy challenges.

These technologies are also transforming other fields. In the aerospace field, deep learning techniques have successfully addressed the challenges in the early detection of small objects [6]. However, there is growing demand for ever faster detection models. This is because detection techniques need to be adapted to perform in real time and exhibit low time consumption. Existing convolutional networks, such as the different versions of YOLO, have allowed significant progress to be made in the area of drone detection for unmanned aircraft. This has significantly impacted public safety, as there has been an increase in the use of drones in society [7].

On the other hand, the integration of AI with micro- and nano-systems has revolutionized healthcare, enabling significant advancements in disease diagnosis, treatment and monitoring. For example, AI-enhanced sensors have demonstrated remarkable accuracy in biomarker detection, facilitating earlier and more precise diagnoses [8]. Additionally, AI-controlled micro- and nanorobots are emerging as promising tools for targeted drug delivery and minimally invasive surgical procedures, improving therapeutic efficacy and reducing side effects [9]. Furthermore, AI has optimized the design and functionality of implantable medical devices, such as pacemakers and glucose sensors, by enabling real-time adaptation to patients’ physiological conditions [10]. These developments highlight the transformative potential of the convergence between AI and micro–nano-systems in enhancing healthcare and improving patients’ quality of life.

Similarly, this integration is generating advances in other industries. The integration of AI with micro-systems has driven significant innovations in the automotive industry, enhancing both the efficiency and safety of modern vehicles. For instance, AI-enhanced micro sensors enable precise real-time detection of obstacles and road conditions, which is essential for the development of autonomous driving technologies and advanced driver-assistance systems [11]. Additionally, nanoengineering-based battery monitoring systems provide detailed data on the performance and status of batteries in electric vehicles, thereby optimizing energy management and extending battery life [12]. AI and nanotechnology are also facilitating the development of lightweight and durable materials for vehicle construction, reducing vehicle weight and improving fuel efficiency [13]. These advancements illustrate how the convergence of AI with micro- and nano-systems in the automotive sector is promoting safer, more efficient and more sustainable mobility.

Currently, one of the challenges in this field is the development of proposals that can be used in limited computational resources, such as embedded systems. Various fields, such as biomedicine and sustainability, have benefited from the development of proposals that maximize the efficiency of their processes [1,14]. To this end, recent research has focused on the development of micro–nano-systems that can execute specific tasks. Figure 2 displays a chart of the main challenges addressed by the development of embedded AI.

These techniques have recently even been integrated into the automotive industry, with a focus on improving users’ interactions with vehicles [15] through methods such as voice recognition [16] or virtual assistants [17]. In addition, it is evident that thanks to these techniques, unmanned aerial vehicles have undergone improvements in their logistics, security and navigation, which demonstrates the cutting-edge nature of these techniques in real scenarios [18,19]. This has allowed for significant advances in autonomous driving vehicles, improving the user experience. Likewise, predictive maintenance has been strengthened in order to prevent failures and reduce costs [20,21].

There has been an increase in the integration of artificial intelligence (AI) and machine learning (ML) in various fields, driving significant advances across multiple application domains. In the energy sector, artificial neural networks (ANNs) have been used in maximum-power point tracking (MPPT) algorithms for photovoltaic systems, improving energy efficiency [22]. Similarly, the automotive industry has leveraged AI-driven image processing methods to improve road safety by accurately detecting distortions in side-view mirrors [23]. In industrial applications, self-adjusting control systems using neural networks have been developed to adapt to varying load conditions, increasing the robustness and efficiency of electric actuators [24]. In the biomedical field, AI models combining U-Net architectures have enabled the accurate segmentation of cerebral blood vessels in angiograms [25] as well as the control of prostheses using electromyographic signals [26]. Furthermore, in agriculture, metaheuristic algorithms integrated with image processing techniques have been implemented to accurately estimate livestock weight [27]. In the field of information security, AI has contributed to the development of robust cryptographic algorithms resistant to side-channel attacks [28]. Finally, advanced object detection models, such as YOLOv3-SPP3 and YOLOv5, have been applied to detect drones and identify anomalies in both clinical and industrial settings, demonstrating the versatility and effectiveness of AI in diverse contexts [29,30].

This Special Issue presents a perspective on the application of artificial intelligence in micro–nano-systems for problem solving. According to the published works, five main areas for the application of this technology have been identified: anthropometry, energy, aircraft, angiography and automotive. According to these works, artificial intelligence models could be made faster and more robust.

## Figures and Tables

**Figure 1:**
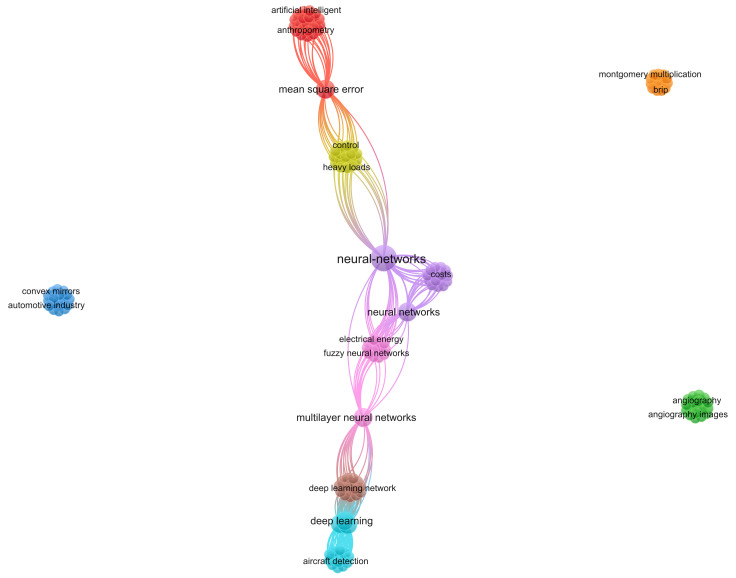
Trends of research on the integration of artificial intelligence with micro–nano-systems.

**Figure 2:**
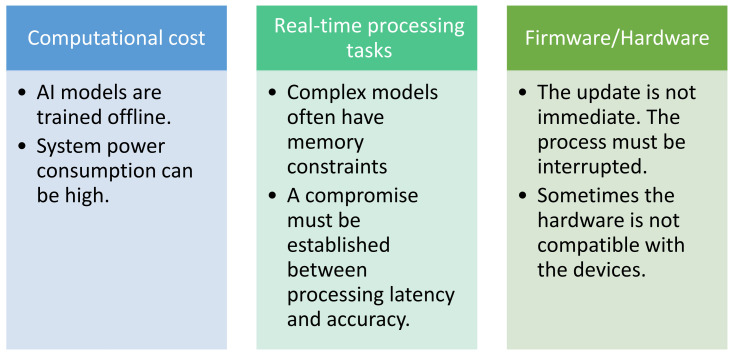
Main challenges in micro–nano-system hardware.

## Data Availability

Not applicable.
